# Supplementary material for: Uncovering uncharacterized binding of transcription factors from ATAC-seq footprinting data
Source: Sci Rep. 2024 Apr 23;14:9275. doi: 10.1038/s41598-024-59989-2 (PMC11039736; doi:10.1038/s41598-024-59989-2)
Supplement: Supplementary file 1 — Supplementary Figures. [file 41598_2024_59989_MOESM1_ESM.docx]

Supplementary data

Uncovering uncharacterized binding of transcription factors from ATAC-seq footprinting data

Hendrik Schultheis^1^, Mette Bentsen^1^, Vanessa Heger^1^, Mario Looso^1,2,*^

**Contents**

[Supplementary Figure 1: Comparison of motif discovery tools 2](#_ma6lgrnpf3p9)

[Supplementary Figure 2: Dux dataset analysis 3](#_ec55rl4qhp56)

[Supplementary Figure 3: Zebrafish dataset analysis. 4](#_rh63bo82rcn7)

[Supplementary Figure 4: Aggregated FPs. 5](#_7cfpt3myd9l9)


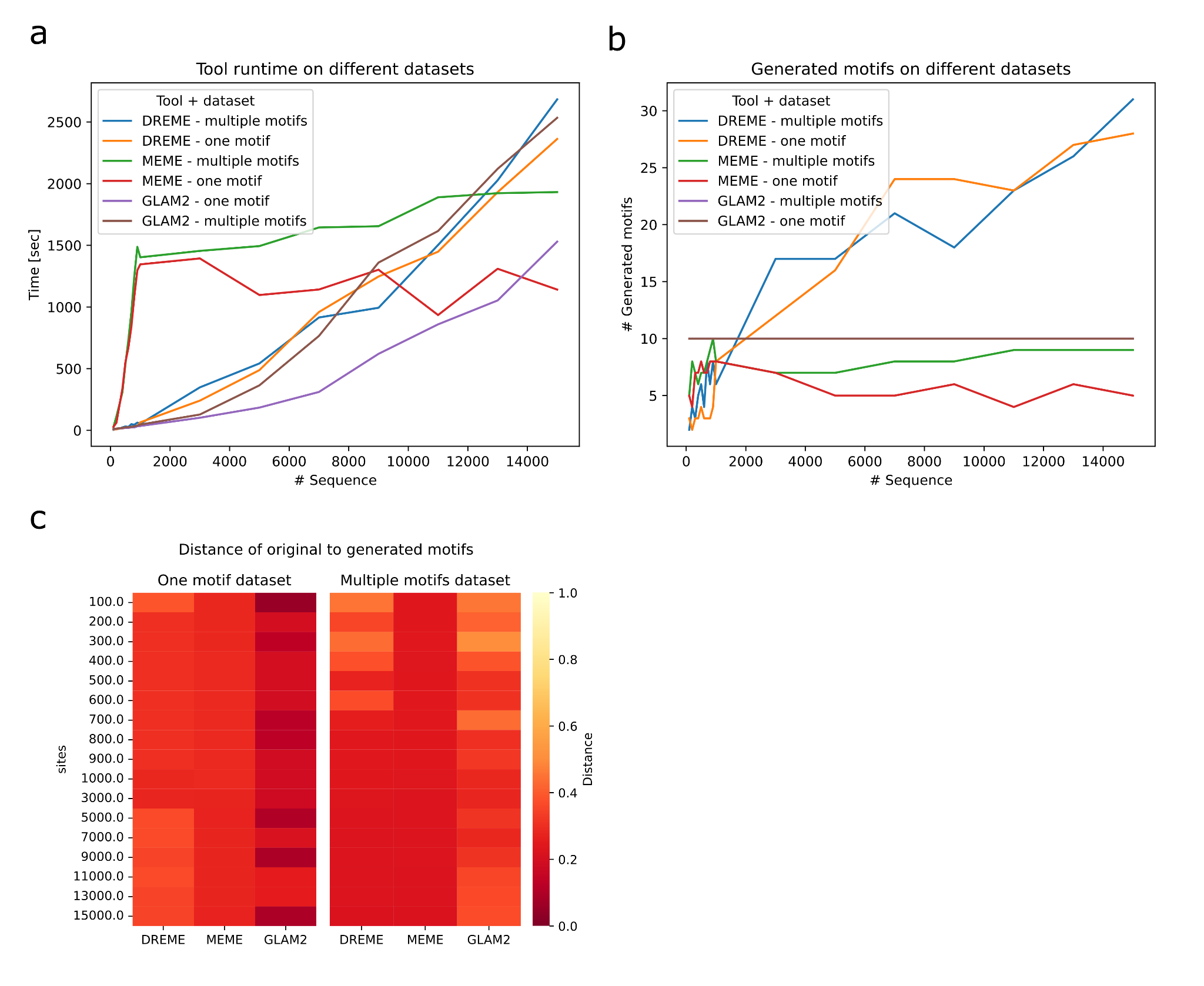


## **Supplementary Figure 1: Comparison of motif discovery tools**

*a) Tool runtime in dependence of number of supplied sequences. Each tool was tested on a dataset containing one motif and a dataset with multiple (four motifs).*

*b) Number of generated motifs in dependence of supplied sequences on the same datasets as in a). GLAM2 is not able to infer the number of motifs based on the data and therefore always generates ten motifs.*

*c) Distance of the closest output motif to the original motif (mean distance for multi-motif datasets). Rows show the number of sites of the given dataset columns show the motif tool. The left side shows runs on datasets with one motif the right are runs on datasets with multiple motifs.*


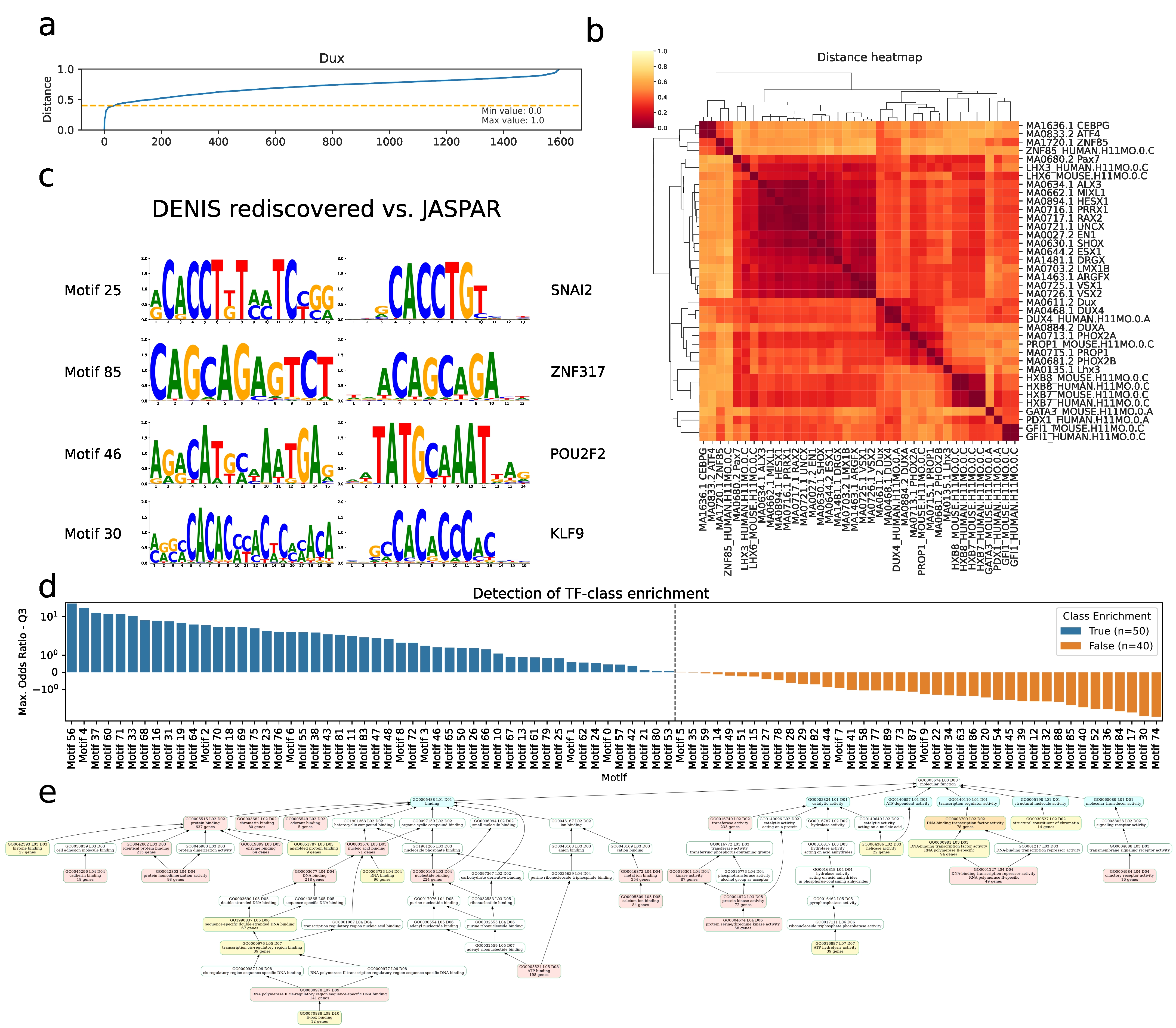


## **Supplementary Figure 2: Dux dataset analysis**

*a) All database motifs sorted by their distance to Dux. Motifs below the threshold are removed for the Dux rediscovery analysis.*

*b) Heatmap of the distances between all motifs below the threshold in a).*

*c) DENIS motifs (left) generated during the Dux differential analysis compared to the closest match of the JASPAR database.*

*d) Categorization of DENIS motifs to show enrichment for TF-classes. Based on the difference between maximum odds ratio and Q3 value (interquartile range) per motif.*

*e) Gene set enrichment analysis of the 2095 genes annotated to motif 10 (Dux) binding sites, using UROPA. Each node describes a GO-Term, with top-level nodes colored blue. Other node colors describe the p-value of a given node with light red denoting high significance (<0.005), light orange medium significance (<0.01), yellow low significance (<0.05), and grey insignificant (>0.05). The GO-Tree was generated using GOATOOLS.*


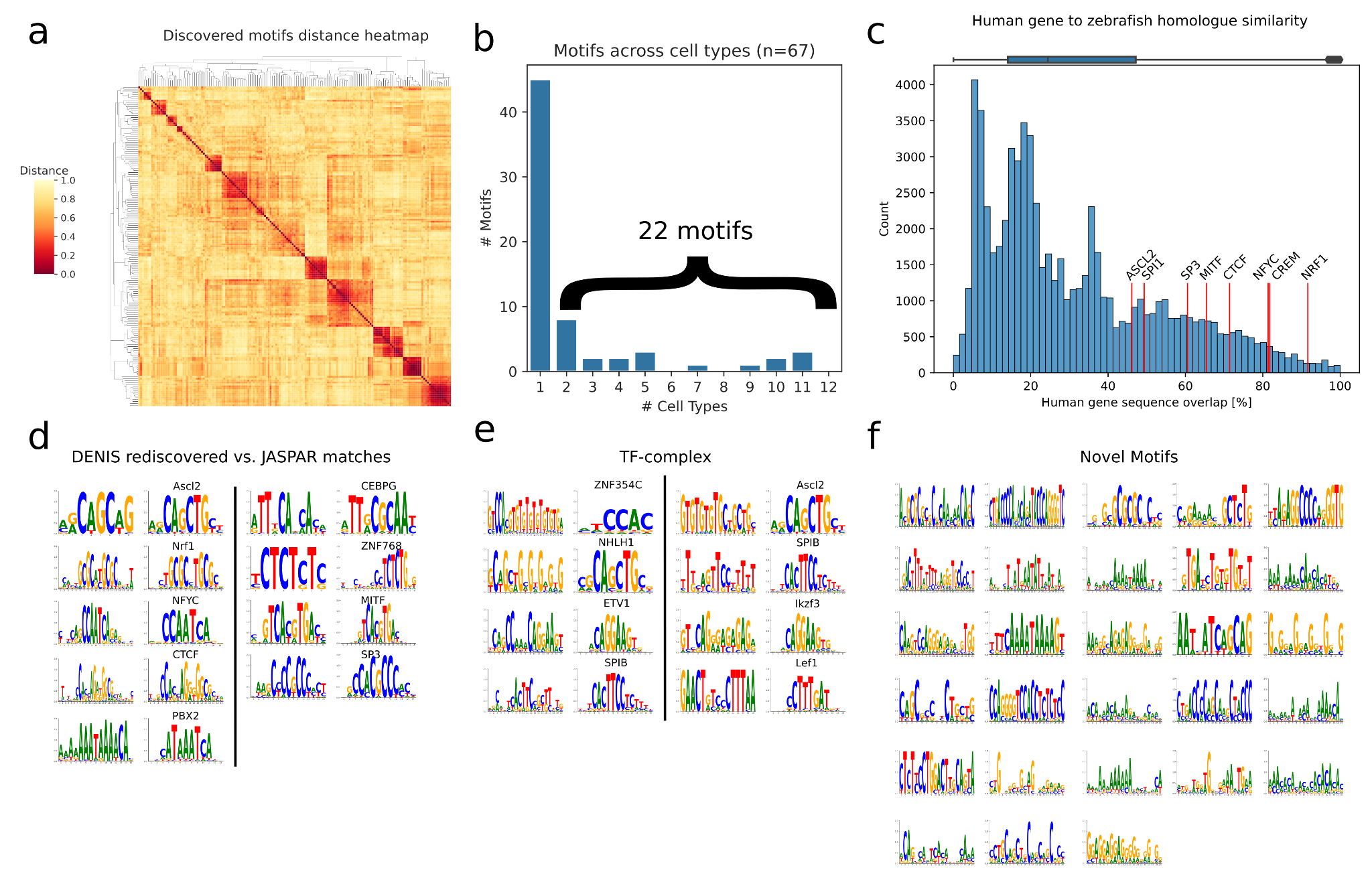


## **Supplementary Figure 3: Zebrafish dataset analysis.**

*a) Distance of DENIS motifs discovered during the WT cell type runs to each other. Motif clusters are combined as they are discovered in multiple cell types.*

*b) The number of motifs counted for their appearance in cell types.*

*c) Distribution of percent sequence overlap of human genes to their zebrafish homologs. The motif of marked genes is rediscovered by the DENIS framework.*

*d-f) Motifs created by the DENIS framework during the zebrafish WT analysis. d) are rediscovered DENIS motifs (left) and their closest JASPAR match (right). e) are assumed to be TF-complex motifs (left) where only part of the motif is known accompanied by their best JASPAR match (right). f) are novel DENIS motifs that did not match a JASPAR motif.*


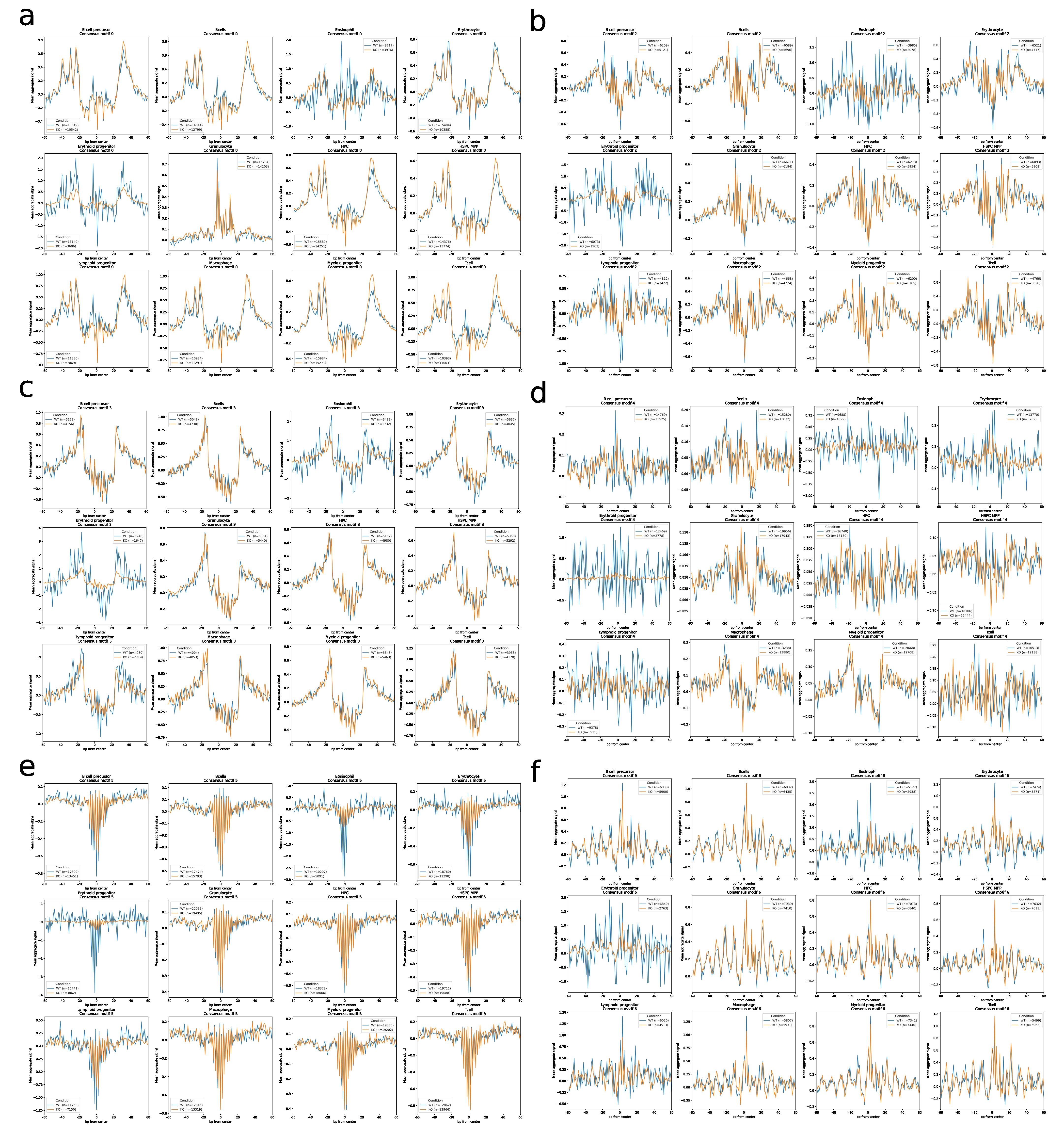


## **Supplementary Figure 4: Aggregated FPs.**

*Aggregated FP of WT (blue) vs. KO (orange) motif positions (created with TOBIAS PlotAggregate) each plot shows the FP for one of the cell types. FPs correspond to consensus motif 0 a), consensus motif 2 b), consensus motif 3 c), consensus motif 4 d), consensus motif 5 e) and consensus motif 6 f).*

**Supplementary Figure 5: Correlation of genomic coverage with the number of identified footprints and generated motifs.**
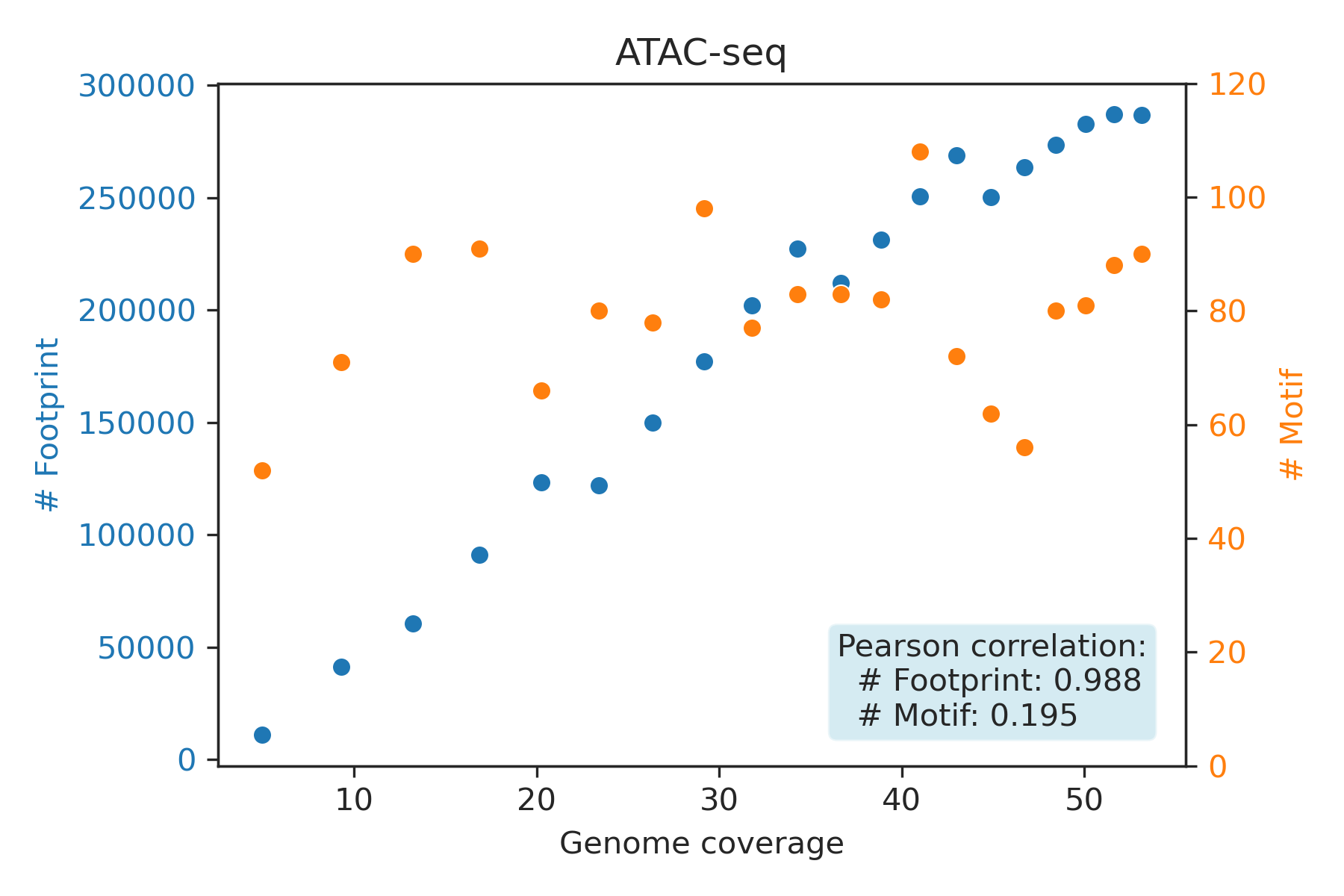


*Random read sampling of the Dux positive condition (Hendrickson et al., 2017) to generate different levels of genome coverage. The number of footprints (left, x-axis) exhibits a strong positive correlation to coverage, whereas the number of motifs displays a weak positive correlation (right, x-axis).*
